# Log *D* versus HPLC derived hydrophobicity: The development of predictive tools to aid in the rational design of bioactive peptoids

**DOI:** 10.1002/bip.23014

**Published:** 2017-06-24

**Authors:** H. L. Bolt, C. E. J. Williams, R. V. Brooks, R. N. Zuckermann, S. L. Cobb, E. H. C. Bromley

**Affiliations:** ^1^ Department of Chemistry Durham University South Road Durham DH1 3LE United Kingdom; ^2^ Department of Physics Durham University South Road Durham DH1 3LE United Kingdom; ^3^ Molecular Foundry, Lawrence Berkeley National Laboratory Berkeley California

## Abstract

Hydrophobicity has proven to be an extremely useful parameter in small molecule drug discovery programmes given that it can be used as a predictive tool to enable rational design. For larger molecules, including peptoids, where folding is possible, the situation is more complicated and the average hydrophobicity (as determined by RP‐HPLC retention time) may not always provide an effective predictive tool for rational design. Herein, we report the first ever application of partitioning experiments to determine the log *D* values for a series of peptoids. By comparing log *D* and average hydrophobicities we highlight the potential advantage of employing the former as a predictive tool in the rational design of biologically active peptoids.

## 1 INTRODUCTION

Peptoids (or *N*‐substituted glycines) are peptidomimetic molecules which are being increasingly investigated for their pharmaceutical properties; as novel anti‐infectives,^1^, ^2^, ^3^, ^4^, ^5^, ^6^, ^7^ biomimetic materials^8^, ^9^ or drug delivery vehicles,^10^, ^11^, ^12^, ^13^ and also for applications within material science.^14^, ^15^, ^16^, ^17^

**Figure 1:**
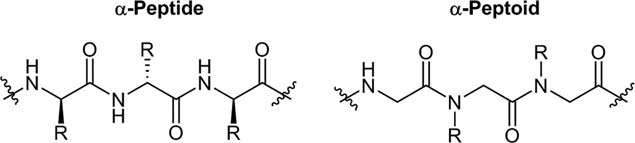
Comparison of a peptide and peptoid backbone

Natural proteins and peptides explore a variety of conformational states from fully stabilised to unfolded and peptoids are no different. Peptoids have been shown to adopt stable and well defined structures in solution, such as the peptoid helix,^18^, ^19^, ^20^ threaded loop conformation,^21^ peptoid nanosheets,^22^, ^23^, ^24^ and nanotubes.^25^ Unlike peptides where regular backbone hydrogen bonding helps to stabilise the secondary structures adopted, peptoids typically rely upon the local steric^26^, ^27^ or electronic effects^28^, ^29^, ^30^ of side chains to help stabilise any secondary structures formed. The positioning of the side chains on the nitrogen of the amide (as opposed to the alpha‐carbon) also renders the backbones of peptoid sequences achiral and the tertiary amides are more easily isomerised between cis and trans conformations than the secondary amides of a peptide (see Figure 1). Overall, this means that the secondary structures adopted by peptoids are influenced heavily by the choice of side‐chains.

Peptoids that contain chiral monomers (e.g. *N*‐(*S*‐1‐phenylethyl) glycine) often appear helical, with a range of different circular dichroism (CD) spectra that are dependent on the side chains used. The chemistry of such structures is well established and these helical states show excellent stability to chemical and thermal denaturation. This has enabled chemists to design helical peptoids with specific functions as it is possible to predict which sequences will form stable helices in solution, for example, mimics of antimicrobial peptides.^1^, ^2^, ^6^, ^7^

To date, there have been few studies that link the secondary structure of peptoids with biophysical properties such as their average hydrophobicity.^31^ Typically, researchers have referred to the reverse‐phase HPLC retention times of compounds as a crude measure of average hydrophobicity and it has been suggested that the hydrophobicity of a given peptoid sequence has a large effect on both toxicity and antimicrobial activity.^1^, ^2^, ^32^

However, in sequences where a peptoids secondary or tertiary structure is dependent on its environment, the hydrophobicity of the folded molecule may be very different, analogously to the exposed hydrophobic area in a folded protein being very different to that seen in the unfolded state. In turn the hydrophobicity of a peptoid in its folded state is likely to play a key role in determining its interactions with biological membranes and hence its biological activity. To study hydrophobicity and peptoid solution structure in more detail we have determined for the first time log *D* values for a series of peptoids (where log *D* represents the distribution coefficient in a buffered aqueous/organic system^33^). Peptoid log *D* values were obtained via partitioning experiments in octanol and phosphate buffered saline (PBS). The data gathered were compared with the average hydrophobicity as determined by reverse‐phase HPLC. In addition, peptoid conformation in solution (both PBS and octanol) was investigated by circular dichroism (CD) spectroscopy. Surprisingly, trends in hydrophobicity were found to differ depending on the method of analysis (i.e. partitioning experiments versus HPLC retention time). It is anticipated that our results will give further insight into the relationship between hydrophobicity and peptoid sequence and that this will in turn help to inform the future rational design of biological active peptoids.

## 2 RESULTS AND DISCUSSION

The peptoids tested in this study were based upon a three‐fold repeat motif *N*x*N*y*N*y and were synthesised on solid phase using the submonomer method of peptoid synthesis (see Table 1)^34^ Sequences were designed with the threefold periodicity to encourage helical character, as previously reported.^1^, ^35^ *N*x represents a hydrophilic monomer with primary amine functionality (either *N*Lys *N*‐(4‐aminobutyl) glycine or *N*ae *N*‐(2‐aminoethyl) glycine, with a 4 carbon chain or 2 carbon chain respectively); *N*y is a hydrophobic residue comprising either *N*spe *N*‐(S‐1‐phenylethyl) glycine or *N*phe *N*‐benzyl glycine, which represent chiral and achiral monomers, respectively. This motif was repeated to give 6, 9, and 12 residue peptoids. Within this library we have the ability to separate the effects of length (via peptoids of identical composition but different lengths), chirality (via peptoids identical except for the substitution of *N*phe for *N*spe) and cationic side chain length (via peptoids identical but for the specific cationic residue used).

**Table 1:** Peptoid library synthesised on solid phase using the sub‐monomer method. All sequences have an N‐terminal free amine and C‐terminal amide group

Peptoid	Sequence	
**1**	(*N*Lys*N*phe*N*phe)_4_	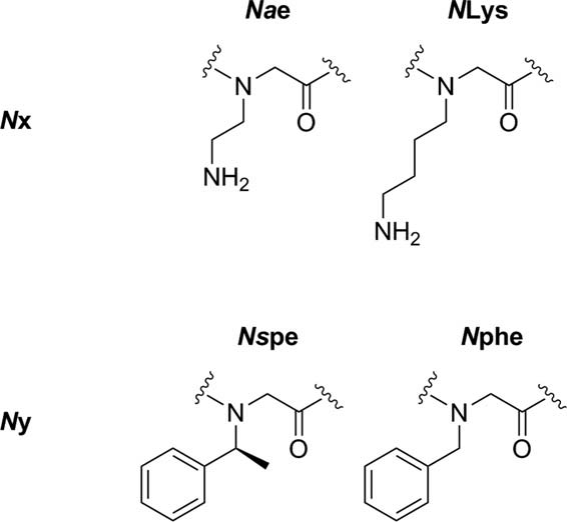
**2**	(*N*Lys*N*phe*N*phe)_3_	
**3**	(*N*Lys*N*phe*N*phe)_2_	
**4**	(*N*Lys*N*spe*N*spe)_4_	
**5**	(*N*Lys*N*spe*N*spe)_3_	
**6**	(*N*Lys*N*spe*N*spe)_2_	
**7**	(*N*ae*N*phe*N*phe)_4_	
**8**	(*N*ae*N*phe*N*phe)_3_	
**9**	(*N*ae*N*phe*N*phe)_2_	
**10**	(*N*ae*N*spe*N*spe)_4_	
**11**	(*N*ae*N*spe*N*spe)_3_	
**12**	(*N*ae*N*spe*N*spe)_2_	

**Figure 2:**
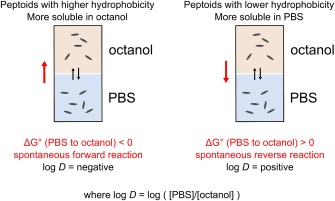
Illustration of the partitioning experiment where during equilibration, peptoids can move between organic and aqueous phases

**Figure 3:**
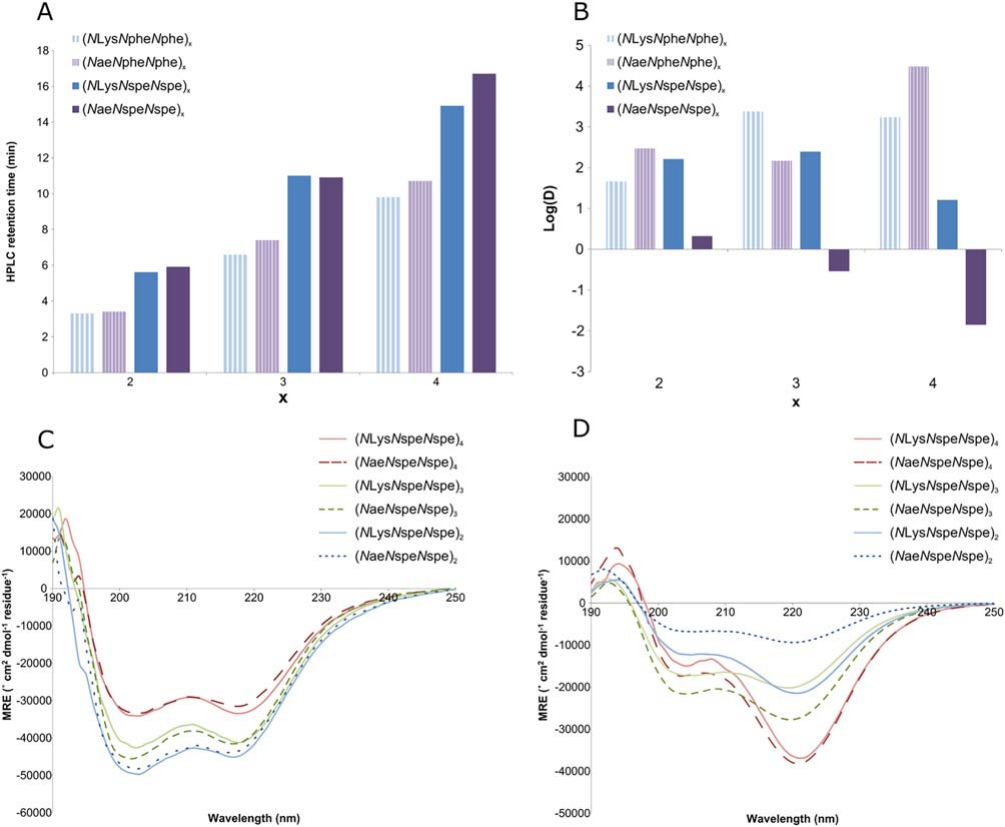
Comparison of average and folded hydrophobicities for peptoid sequences with different lengths, containing either chiral (*N*spe) or achiral (*N*phe) residues and containing either shorter (*N*ae) or longer (*N*Lys) positive side chains: (A) reverse phase HPLC retention times and (B) log *D* as calculated from partition experiments. Also, a comparison of CD spectra for all of the chiral peptoids: (C) in PBS and (D) in octanol

To examine the average hydrophobicity of the peptoids, the retention time for from reverse‐phase HPLC was determined on a C18 column using acetonitrile and water, with 0.1% TFA (see Figure 3A). To find log *D* values, partitioning experiments were carried out, as illustrated by Figure 2. A peptoid solution (between 10 and 100 µM) in PBS was added to an equal volume of octanol and the system allowed to equilibrate under gentle agitation for ∼150 h (as 48 h was not found to be sufficient for the system to reach equilibrium). At this point it is assumed that the peptoid has partitioned between these two phases, such as to minimise its free energy.

A combination of UV spectrometry and dynamic light scattering were used to determine that no aggregation was taking place in either phase (see Supporting Information). The peptoid concentrations of both phases were then determined using UV–Vis spectroscopy and the logarithm of the ratio of concentration in PBS over concentration in octanol (log *D*) was calculated (Figure 3B).

Often, reverse‐phase HPLC retention times of compounds are used as a crude measure of average hydrophobicity to rationalise behaviour (e.g. antibacterial properties). 1, 2, 36 The HPLC retention times obtained herein indicate that the peptoids become more hydrophobic as the overall length increases (Figure 3A).There is also an increase in hydrophobicity caused by switching from the achiral *N*phe monomer to the chiral *N*spe monomer (e.g. compare Peptoid **1** (*N*Lys*N*phe*N*phe)_4_ and Peptoid **4** (*N*Lys*N*spe*N*spe)_4_ in Figure 3A). It has previously been suggested that this change may be due to the extra alpha‐CH_3_ group present in *N*spe monomer adding more hydrophobicity to the peptoid. However, it may be that this is a too simplistic an explanation given that there is actually a small decrease in the hydrophobicity when the *N*Lys monomer is replaced in a sequence with the shorter alkyl chained *N*ae unit (e.g. compare Peptoids **1** (*N*Lys*N*phe*N*phe)_4_ and **7** (*N*ae*N*phe*N*phe)_4_ in Figure 3A).

The pH of our HPLC system (solvent/water + 0.1% TFA) is around pH 2.2, whereas the partitioning is carried out in PBS at approximately pH 7.4. Although these pH values are different, the basic amino‐functionalised side chains will be protonated in both cases. However, in different peptoid sequences, with acidic side chains or those with a lower p*K*a, the discrepancy between HPLC pH conditions and partitioning may become more important. We believe that the partitioning experiments will be very useful in these cases, as the hydrophobicity measured from log *D* values may be more representative of biological conditions. From the log *D* hydrophobicity data in Figure 3B, it can be seen that the log *D* values are similar for all peptoids except for the 9 and 12mers with the combination of both *N*ae and *N*spe (Peptoids **10** (*N*ae*N*spe*N*spe)_4_ and **11** (*N*ae*N*spe*N*spe)_3_). Peptoids **10** and **11** are the only peptoids in the library to show significant movement into the hydrophobic phase (e.g. they have negative log *D* values). It can also be seen from comparison of Peptoids **10** and **11** that, increasing the sequence length gives rise to an increase in partitioning to the hydrophobic phase (i.e. Peptoid **10** partitions into the octanol layer more that Peptoid **11**).

The dramatic differences in the two descriptions of hydrophobicity indicate that the folding of peptoids in both the aqueous and hydrophobic phases is important to their biophysical properties. In particular, it is clear that the difference in energy between the folded state adopted in PBS and the folded state adopted in octanol is greater when the peptoid is longer, there are chiral residues present, and the positive side chains are shorter (i.e. *N*ae preferred over *N*Lys). All of the peptoids gave a significant CD signal and hence were found to be adopting helical folded conformations in both PBS and octanol (see Figure 3C,D).

The peptoids in PBS show a change in the shape of the CD spectra as the length increases, perhaps indicating that the lowest energy conformation changes as the peptoid becomes longer. The CD spectra of the peptoids in octanol show a different shape to those in PBS and again exhibits a variation with peptoid length. Again this indicated a difference in the details of the helical structure being adopted. For the two longest chiral peptoids, Peptoid **4** (*N*Lys*N*spe*N*spe)_4_ and **10** (*N*ae*N*spe*N*spe)_4_ there is no difference in either PBS or octanol as a function of cationic side chain length. The large difference in folded hydrophobicity between these two peptoids must therefore stem from a difference in energy of the same folded state when adopted with short (*N*ae) or long (*N*Lys) cationic side chains. We cannot determine from these data whether the long side chain stabilises the aqueous folded state or destabilised the hydrophobic folded state or if both are occurring. A likely hypothesis for this difference is that the folded conformation may enable increased shielding of the positive charge from the solvent in the short chained case that would be energetically unfavourable in PBS and energetically favourable in octanol.

To consider how log *D* could be used as a tool to help rationalise and predict the antimicrobial properties of peptoids we selected Peptoids **4** and **10** as they have similarly HPLC retention times (14.9 and 16.7 min respectively), the same overall charge (+4) but very different log *D* values (+1.21 and −1.85, respectively). As Table 2 highlights, comparison of the biological activity of Peptoids **4** and **10** shows that in some cases (*L. mexicana* promastigotes and representative gram positive bacteria like *S. aureus*) similar biological activities are seen for both. If analyzing the biological data purely based on the HPLC retention times of Peptoids **4** and **10** then this is what would be expected. However, when screening against other microbes which have different membrane compositions, the hydrophobicity as determined by log *D* may prove to be more significant in predicting activity. For example, against axenic *L. mexicana* amastigotes, the causative agent of cutaneous leishmaniasis, Peptoid **10** has a greater biological activity compared with Peptoid **4** (Table 2, ED_50_ 17 compared with >100 µM). Similarly, the activity of the two peptoids against *E. coli* differs significantly (Table 2). These differences in activity cannot be rationalised in terms of HPLC retention time, but the clear difference in the log *D* values does offer a route by which to probe the link between physical properties and biological activity in more detail. It also highlights that analogous to antimicrobial peptides the biological activity against a specific microbe is likely to be dependent on a given peptoids ability to fold in solution.

**Table 2:** A comparison of the biological activity of the peptoid library with the analytical HPLC retention times and log *D* values. Gradient: 0–100% solvent B over 30 min at 220 nm, conditions as in the Supporting Information. ED_50_ represents the median effective dose and MIC the minimum inhibitory concentration. All biological screening was done in triplicate on a minimum of two separate occasions to ensure a robust data set was collected, using protocols as in the Supporting Information^6^, ^7^,^[37]^

			**ED_**50**_ (µM)**		**MIC (µM)**			
**Peptoid**	**Sequence**	**MW (g mol^**−1**^)**	***L. mex prom*.**	***L. mex ama*.**	***E. coli***	***S. aureus***	**HPLC RT (min)**	**log *D***
**4**	(*N*Lys*N*spe*N*spe)_4_	1819.32	8	>100	25	2	14.9	1.21
**10**	(*N*ae*N*spe*N*spe)_4_	1707.11	7	17	>100	2	16.7	−1.85

## 3 CONCLUSION

In this study we have measured for the first time the log *D* values for a series of peptoids that mimic antimicrobial peptides via a partition experiment between PBS buffer and octanol. The log *D* values determined provide a measure of the hydrophobicity of the folded state of a peptoid in solution. It was noted that hydrophobicity as determined by log *D* can be significantly different to the hydrophobicity as determined by HPLC retention time. By looking at the biological activity of two peptoids with similar HPLC retention times but different log *D* values we were able to demonstrate that hydrophobicity, as measured by log *D*, could provide a new method to analyze the biological properties of membrane targeting peptoids. We are currently in the process of collating a larger data set to further probe the potential application of using log *D* values as a predictive tool to enable the rational design of biologically active peptoids in the future.

## 4 EXPERIMENTAL

### 4.1 Materials and reagents

Abbreviations for reagents are as follows: tert‐butoxycarbonyl (Boc); 9‐fluorenylmethoxylcarbonyl (Fmoc); trifluoroacetic acid (TFA); triisopropylsilyl (TIPS); *N*,*N*‐dimethylformamide (DMF); *N*,*N*‐diisopropylcarbodiimide (DIC); dimethylsulphoxide (DMSO); bromoacetic acid (BrAA). Solvents and reagents were purchased from commercial sources and used without further purification unless otherwise noted.

### 4.2 Peptoid synthesis

Automated peptoid synthesis using an Aapptec Apex 396 synthesiser. Fmoc‐protected Rink Amide resin (0.1 mmol, loading 0.54 mmol g^−1^) was swollen in DMF (2 mL, 2 min, 475 rpm at RT) and deprotected with 4‐methylpiperidine (20% in DMF v/v, 1 mL for 1 min, 475 rpm at RT; then 2 mL for 12 min, 475 rpm at RT). The resin was treated with bromoacetic acid solution (1 mL, 0.6M in DMF) and DIC (0.18 mL, 50% v/v in DMF) for 20 min at 475 rpm, RT. The resin was washed with DMF (2 mL DMF for 1 min at 475 rpm, × 5) before the desired amine sub‐monomer was added (1 mL, 1.5M in DMF) and shaken for 60 min at 475 rpm. The resin was washed again with DMF (2 mL DMF for 1 min at 475 rpm, × 5) and the acetylation and amine displacement steps were repeated until the desired sequence was achieved. The resin washed with dichloromethane and peptoids cleaved off the resin using a TFA cleavage cocktail (4 mL; TFA:TIPS:H_2_O, 95:2.5:2.5) for 30–60 min on an orbital shaker at 250 rpm, RT. The cocktail was filtered from the resin and evaporated in vacuuo and the resulting residue precipitated in diethyl ether (∼20 mL). The crude peptoid was obtained via centrifugation (15 min, 4000 rpm, 5°C) and the ether layer decanted to yield the crude product as a powder. Peptoids were lyophilised before purification by semi‐preparative RP‐HPLC.

Preparative RP‐HPLC was performed with a semi‐preparative Perkin Elmer Series 200 lc pump fitted with a 785A UV/Vis detector using a SB‐Analytical ODH‐S optimal column (250 mm × 10 mm, 5 µm); flow rate 2 mL min^−1^; *λ* = 250 nm, typical linear gradient elution 0–50% of solvent B over 60 min (A = 0.1% TFA in 95% H_2_O and 5% MeCN, B = 0.1% TFA in 5% H_2_O and 95% MeCN). Analytical RP‐HPLC was performed with a Perkin Elmer Series 200 LC pump fitted with a Series 200 UV/Vis detector using a SB‐Analytical ODH‐S optimal column (100 mm × 1.6 mm, 3.5 µm); flow rate 1 mL min^−1^; *λ* = 220 nm, linear gradient elution 0–100% of solvent B over 30 min (A = 0.05% TFA, 95% H_2_O, 5% MeCN, B = 0.03% TFA, 5% H_2_O, 95% MeCN).

Peptoids were characterised by accurate LC‐MS (QToF mass spectrometer and an Acquity UPLC from Waters Ltd) using an Acquity UPLC BEH C8 1.7μm (2.1 mm × 50 mm) column with a flow rate of 0.6 mL min^−1^ and a linear gradient of 5–95% of solvent B over 3.8 min (A = 0.1% formic acid in H2O, B = 0.1% formic acid in MeCN). Peptide identities were also confirmed by MALDI‐TOF mass spectra analysis (Autoflex II ToF/ToF mass spectrometer Bruker Daltonik GmBH) operating in positive ion mode using an α‐cyano‐4‐hydroxycinnamic acid (CHCA) matrix. Data processing was done with MestReNova Version 8.1.

### 4.3 Biophysical characterisation

Peptoids were dissolved at concentrations between 10 and 300 µM in either Phosphate Buffered Saline or 1‐Octanol. Exact concentrations were measure using UV spectrometry (Shimadzu UV‐3600) using the phenylalanine‐like peak centred at 258 nm and a molar extinction coefficient of 195 M^−1^ cm^−1^ per residue. It was necessary to subtract baselines and the influence of the peptoid backbone absorption at lower wavelengths to get accurate concentration data.

Partition experiments were carried out by putting 450 µL of octanol in contact with 450 µL of PBS, which contained between 10 and 100 µL of peptoid. Each peptoid was measured in triplicate. The samples were allowed to equilibrate under gentle agitation for ∼150 h (as 48 h was not found to be sufficient for the system to reach equilibrium). After this point samples were taken from the PBS half and the octanol half and diluted to produce sufficient volume for spectroscopy. The concentration of peptoid remaining in the PBS and the octanol was measured individually using the phenylalanine peak as before. From these concentrations the ratio Kv of concentration in PBS to concentration in octanol was calculated along with the free energy of insertion into octanol ΔG = RTlnK_v_. All PBS solutions were checked for aggregation both by inspection of the UV spectra to look for scattering effects and by measuring particle size using dynamic light scattering (DLS) (Malvern Zetasizer Nano). DLS data were collected at higher peptoid concentration, between 100 and 300 µM to increase the signal to noise. Samples of peptoid in octanol were also tested for those peptoids that partitioned significantly into octanol. It was further noted that no material was seen to aggregated during partition experiments as the final amount of peptoid did not change from the initial amount.

Circular Dichroism spectra were collected for all the peptoids containing the chiral *N*spe residue (Jasco J‐1500 CD spectrophotometer) using 1 mm path length and 3 nm bandwidth. The peptoid was measured at concentrations around 30 µM with all spectra being reported in Molar Ellipticity.

All of the peptoids were checked at high concentration (∼500 µM) in PBS for indicators of aggregation using dynamic light scattering. Due to the increase in intensity of scattering with size, the presence of a signal at small hydrodynamic diameters indicates the overwhelming majority of the sample is present in the smallest peak.

## Supporting information

Supporting Information
