# Supplementary material for: Log D versus HPLC derived hydrophobicity: The development of predictive tools to aid in the rational design of bioactive peptoids
Source: Biopolymers. 2017 Jun 24;108(4):e23014. doi: 10.1002/bip.23014 (PMC5519928; doi:10.1002/bip.23014)
Supplement: Supplementary file 1 — Supporting Information [file BIP-108-na-s001.docx]

**LogD *versus* HPLC derived hydrophobicity: The development of predictive tools to aid in the rational design of bioactive peptoids**

*Supporting information for*

H. L. Bolt^b^, C.E.J Williams^a^, R.V. Brooks,^a^ , R.N. Zuckermann^c^, S. L. Cobb*^*,b^* and E.H.C Bromley*^*,a^*

^a^ Department of Physics, Durham University, South Road, Durham, DH1 3LE, UK. *E-mail: [e.h.c.bromley@durham.ac.uk](mailto:e.h.c.bromley@durham.ac.uk)

^b^ Biophysical Sciences Institute, Department of Chemistry, Durham University, South Road, Durham, DH1 3LE, UK. *E-mail: [s.l.cobb@durham.ac.uk](mailto:s.l.cobb@durham.ac.uk)

^c^ Molecular Foundry, Lawrence Berkeley National Laboratory, Berkeley, California, USA

*E-mail:* [rnzuckermann@lbl.gov](mailto:rnzuckermann@lbl.gov)

**Contents**

[1. Synthesis Procedures 3](#_Toc457494592)

[1.1. Materials and Reagents 3](#_Toc457494593)

[1.2. Peptoid Synthesis 3](#_Toc457494594)

[2. Biophysical Characterisation 5](#_Toc457494595)

[2.1 Dynamic Light Scattering Data 6](#_Toc457494596)

[3. Biological Assays 12](#_Toc457494597)

[4. Characterisation 14](#_Toc457494598)

[4.1. Characterisation of building blocks and peptoids used in this study 14](#_Toc457494599)

[4.2. Accurate Mass Data 15](#_Toc457494600)

[4.3. Representative Analytical RP-HPLC 16](#_Toc457494601)

[4.4. Chemical and Physical Data for Peptoid Library 19](#_Toc457494602)

[4.5. LC-MS Spectra for Peptoid Library 20](#_Toc457494603)

# Synthesis Procedures

## Materials and Reagents

Abbreviations for reagents are as follows: *tert*-butoxycarbonyl (Boc); 9-fluorenylmethoxylcarbonyl (Fmoc); trifluoroacetic acid (TFA); triisopropylsilyl (TIPS); *N,N*-dimethylformamide (DMF); *N,N*-diisopropylcarbodiimide (DIC); dimethylsulphoxide (DMSO); bromoacetic acid (BrAA). Solvents and reagents were purchased from commercial sources and used without further purification unless otherwise noted.

## Peptoid Synthesis

Automated peptoid synthesis using an Aapptec Apex 396 synthesiser. Fmoc-protected Rink Amide resin (0.1 mmol, loading 0.54 mmol g^-1^) was swollen in DMF (2 mL, 2 min, 475 rpm at RT) and deprotected with 4-methylpiperidine (20% in DMF *v*/*v*, 1 mL for 1 min, 475 rpm at RT; then 2 mL for 12 min, 475 rpm at RT). The resin was treated with bromoacetic acid solution (1 mL, 0.6M in DMF) and DIC (0.18 mL, 50% v/v in DMF) for 20 min at 475 rpm, RT. The resin was washed with DMF (2 mL DMF for 1 min at 475 rpm, x 5) before the desired amine sub-monomer was added (1 mL, 1.5M in DMF) and shaken for 60 mins at 475 rpm. The resin was washed again with DMF (2 mL DMF for 1 min at 475rpm, x 5) and the acetylation and amine displacement steps were repeated until the desired sequence was achieved. The resin washed with dichloromethane and peptoids cleaved off the resin using a TFA cleavage cocktail (4 ml; TFA:TIPS:H_2_O, 95:2.5:2.5) for 30-60 min on an orbital shaker at 250 rpm, RT. The cocktail was filtered from the resin and evaporated *in vacuuo* and the resulting residue precipitated in diethyl ether (~20 ml). The crude peptoid was obtained via centrifugation (15 mins, 4,000 rpm, 5 ˚C) and the ether layer decanted to yield the crude product as a powder. Peptoids were lyophilised before purification by semi-preparative RP-HPLC.

Preparative RP-HPLC was performed with a semi-preparative Perkin Elmer Series 200 lc pump fitted with a 785A UV/Vis detector using a SB-Analytical ODH-S optimal column (250 × 10 mm, 5 µm); flow rate 2 ml min^−1^; λ = 250 nm, typical linear gradient elution 0-50% of solvent B over 60 min (*A* = 0.1% TFA in 95% H_2_O and 5% MeCN, *B* = 0.1% TFA in 5% H_2_O and 95% MeCN). Analytical RP-HPLC was performed with a Perkin Elmer Series 200 LC pump fitted with a Series 200 UV/Vis detector using a SB-Analytical ODH-S optimal column (100 × 1.6 mm, 3.5 µm); flow rate 1 ml min^−1^; λ = 220 nm, linear gradient elution 0-100% of solvent B over 30 min (*A* = 0.05% TFA, 95% H_2_O, 5% MeCN, *B* = 0.03% TFA, 5% H_2_O, 95% MeCN).

Peptoids were characterised by accurate LC-MS (QToF mass spectrometer and an Acquity UPLC from Waters Ltd.) using an Acquity UPLC BEH C8 1.7μm (2.1mm × 50mm) column with a flow rate of 0.6 ml min^-1^ and a linear gradient of 5-95% of solvent B over 3.8 min (*A* = 0.1% formic acid in H_2_O, *B* = 0.1% formic acid in MeCN). Peptide identities were also confirmed by MALDI-TOF mass spectra analysis (Autoflex II ToF/ToF mass spectrometer Bruker Daltonik GmBH) operating in positive ion mode using an α-cyano-4-hydroxycinnamic acid (CHCA) matrix. Data processing was done with MestReNova Version 8.1.

*Figure S1*. The submonomer method of peptoid synthesis on solid phase; [i] swelling and deprotection of resin; [ii] acylation using bromoacetic acid and DIC in DMF; [iii] displacement step (primary amine in DMF); [iv/v] successive cycles of acylation and displacement; [vi] acidic TFA cleavage of product from resin.

# Biophysical Characterisation

Peptoids were dissolved at concentrations between 10 and 300 µM in either Phosphate Buffered Saline or 1-Octanol. Exact concentrations were measure using UV spectrometry (Shimadzu UV-3600) using the phenylalanine-like peak centred at 258 nm and a molar extinction coefficient of 195 M^-1^ cm^-1^ per residue. It was necessary to subtract baselines and the influence of the peptoid backbone absorption at lower wavelengths in order to get accurate concentration data.

Partition experiments were carried out by putting 450 µL of octanol in contact with 450 µL of PBS, which contained between 10 and 100 µL of peptoid. Each peptoid was measured in triplicate. The samples were allowed to equilibrate under gentle agitation for ~150 hours (as 48 hours was not found to be sufficient for the system to reach equilibrium). After this point samples were taken from the PBS half and the octanol half and diluted to produce sufficient volume for spectroscopy. The concentration of peptoid remaining in the PBS and the octanol was measured individually using the phenylalanine peak as before. From these concentrations the ratio K_v_ of concentration in PBS to concentration in octanol was calculated along with the free energy of insertion into octanol $\Delta G=RT\ln K_{v}$. All PBS solutions were checked for aggregation both by inspection of the UV spectra to look for scattering effects and by measuring particle size using dynamic light scattering (DLS) (Malvern Zetasizer Nano). DLS data were collected at higher peptoid concentration, between 100 and 300 µM to increase the signal to noise. Samples of peptoid in octanol were also tested for those peptoids that partitioned significantly into octanol. It was further noted that no material was seen to aggregated during partition experiments as the final amount of peptoid did not change from the initial amount.

Circular Dichroism spectra were collected for all the peptoids containing the chiral *N*Spe residue (Jasco J-1500 CD spectrophotometer) using 1 mm pathlength and 3 nm bandwidth. The peptoid was measured at concentrations around 30 µM with all spectra being reported in Molar Ellipticity.

All of the peptoids were checked at high concentration (~500 µM) in PBS for indicators of aggregation using dynamic light scattering. Due to the increase in intensity of scattering with size, the presence of a signal at small hydrodynamic diameters indicates the overwhelming majority of the sample is present in the smallest peak.

## 2.1 Dynamic Light Scattering Data

**Peptoid 1 (*N*Lys*N*phe*N*phe)_4_**

**Peptoid 2 (*N*Lys*N*phe*N*phe)_3_**

**Peptoid 3 (*N*Lys*N*phe*N*phe)_2_**

**Peptoid 7 (*N*ae*N*phe*N*phe)_4_**

**Peptoid 8 (*N*ae*N*phe*N*phe)_3_**

**Peptoid 9 (*N*ae*N*phe*N*phe)_2_**

**Peptoid 4 (*N*Lys*N*spe*N*spe)_4_**

**Peptoid 5 (*N*Lys*N*spe*N*spe)_3_**

**Peptoid 6 (*N*Lys*N*spe*N*spe)_2_**

**Peptoid 10 (*N*ae*N*spe*N*spe)_4_**

**Peptoid 11 (*N*ae*N*spe*N*spe)_3_**

**Peptoid 12 (*N*ae*N*spe*N*spe)_2_**

Those peptoids that substantially partitioned into the octanol during the partitioning experiment were checked at high concentration (~500 µM) in Octanol for indicators of aggregation using dynamic light scattering. Due to the increase in intensity of scattering with size, the presence of a signal at small hydrodynamic diameters indicates the overwhelming majority of the sample is present in the smallest peak.

**Peptoid 4(*N*Lys*N*spe*N*spe)_4_**

**Peptoid 10 (*N*ae*N*spe*N*spe)_4_**

**Peptoid 5 (*N*Lys*N*spe*N*spe)_3_**

**Peptoid 11 (*N*ae*N*spe*N*spe)_3_**

# Biological Assays

**Cytotoxicity Assays with *Leishmania mexicana* M379 Promastigotes and Amastigotes**

Leishmania mexicana (M379) promastigote parasites were maintained at 26 °C in Schneider's Insect medium (Sigma-Aldrich) supplemented with heat-inactivated foetal bovine sera (FBS, 15%; Biosera Ltd). Cells were counted using a Neubauer Improved Haemocytometer. Promastigotes were transformed into axenic amastigotes by a pH and temperature shift as previously described.5 A culture of recently transformed (three days) promastigotes in the late log phase was transferred into Schneider's Insect medium supplemented with 20% heat-inactivated FBS (pH 5.5) at 5 x 105 parasites/mL. After 6 days, the parasites were in the metacyclic stage and used for transformation to amastigote-like forms by transfer in the same medium at 32 °C at 5 x 105 parasites/mL. After additional 5–7 days, the parasites should be in the amastigote stage and be ready for cytotoxicity studies and infections.

Cytotoxicity analyses were performed in 96-well plates (Costar, Fisher Scientific) using alamarBlue (Invitrogen) for cell viability detection as previously described.6 Promastigote and amastigote L. mexicana were pre-incubated with the compounds in triplicate (5 mM stock solutions in DMSO; Amphotericin B was used as a positive control; untreated parasites with DMSO as a negative control) in 50 µL of the corresponding media at 4 x 106 mL-1 for 1 hour. Afterwards, 40 µL were removed from each well before the addition of 90 µL of the corresponding media, followed by incubation for 24 hours at 4 x 105 mL-1. Then, 10 µL alamarBlue solution (Invitrogen) was added to each well for an incubation of 4 hours prior to assessing cell viability using a fluorescent plate reader (Biotek; Ex 560 nm / Em 600 nm). To investigate the effects of serum on the efficacy of the peptoids, the assay described above was modified using serum-free medium for the pre-incubation time. For these assays, the parasites were washed three times in serum-free medium before adding them to the compound solutions. All of the experiments described above were carried out on a minimum of two separate occasions to ensure a robust data set was collected.

**Antibacterial Minimum Inhibitory Concentration Determination**

Bacterial cultures were prepared by streaking the bacterial strain from a 4°C- or frozen stock culture to single colonies onto agar plates (containing the appropriate antibiotic for strains carrying antibiotic resistance plasmids) with an inoculation loop, followed by overnight incubation at 37 °C.

Primary overnight (o/n) cultures were prepared by inoculating a single colony from a streak plate into 5 mL of LB broth in a screw-capped culture tube, that was placed on a tube rotator at 37 °C (or 30 °C for sensitive strains) for overnight incubation. Before the usage of stock cultures the strains genotype were checked. Primary stocks were stored at 4°C for up to 6 weeks and always replaced from fresh colonies.

Fresh cultures used for experiments contained 0.1-0.5 mL o/n culture diluted into 8 mL of fresh medium in a standard test tube. Placed in a shaking water bath or on a tube rotator the bacteria were grown at the required temperature to the required cell density. Frozen stock cultures used for long term storage that can remain viable for 10 years were prepared by mixing 2.5 mL fresh o/n culture with 1.5 mL sterile 80% glycerol in a 5 mL sterile blood tube and stored at -20 °C.

MIC values were attained according to the previously described protocol4 and were conducted in 96-well plates (Costar, Fisher Scientific). Using the pre-prepared cultures, an innoculum density of 0.07 at 650 nm was set by comparing the absorbance with 0.5 MacFarland standard (240 μM BaCl2 in 0.18 M H2SO4). The innoculum was then diluted down ten-fold with Iso-sensitest broth (Oxoid, ThermoScientific) before use. Peptoids were initially dissolved in DMSO (5 mM) and then diluted further with Iso-sensitest broth to achieve a concentration range of 4 – 200 µM using 1 : 2 serial dilutions. 50 μl of innoculum and 50 μL of peptoid solution were added to each test well (final concentration range of 2 – 100 µM). Positive control containing only innoculum and Iso-sensitest broth; negative control containing inoculum and serial dilutions of ampicillin (from 250 microgram/mL to 2 microgram per mL); DMSO control containing innoculum and serial dilutions of DMSO; and Iso-sensitest broth were used as a sterile control. MIC was defined as the lowest concentration which completely inhibited bacterial growth after incubation at 38 °C for 16 hours with shaking. Quantitative data was attained from absorbance values using a microplate reader.

# Characterisation

## Characterisation of building blocks and peptoids used in this study

The following Table S1 shows the amine sub-monomers used to synthesize the peptoids described in this paper.

| Monomer | Chemical Structure | Amine Sub-monomer |
| --- | --- | --- |
| *N*Lys  *N*-(4-aminobutyl) glycine |  | *N*-Boc-1,4-diaminobutane |
| *N*ae  *N*-(4-aminoethyl) glycine |  | *N*-Boc-1,4-diaminoethane |
| *N*phe  *N*-(phenylmethyl) glycine |  | benzylamine |
| *N*spe  N(*S*-phenylethyl) glycine |  | *(S)*-(−)-α-Methylbenzylamine |

*Table S1. The abbreviations used for the peptoid monomers synthesised in this study, and the amines that they are derived from.*

## Accurate Mass Data

Accurate mass data for the peptoid library are shown in *Table S2.*

|  | Sequence | Molecular Formula | Mass Calculated  *[M+2H]^2+^* | Mass Observed  *[M+2H]^2+^* |
| --- | --- | --- | --- | --- |
| 1 | (*N*Lys*N*phe*N*phe)_4_ | C_96_H_123_N_17_O_12_ | 853.9847 | 853.9848 |
| 2 | (*N*Lys*N*phe*N*phe)_3_ | C_72_H_93_N_13_O_9_ | 642.8688 | 642.8666 |
| 3 | (*N*Lys*N*phe*N*phe)_2_ | C_48_H_63_N_9_O_6_ | 431.7529 | 431.7513 |
| 4 | (*N*Lys*N*spe*N*spe)_4_ | C_104_H_139_N_17_O_12_ | 910.0473 | 910.0494 |
| 5 | (*N*Lys*N*spe*N*spe)_3_ | C_78_H_105_N_13_O_9_ | 684.9157 | 684.9142 |
| 6 | (*N*Lys*N*spe*N*spe)_2_ | C_52_H_71_N_9_O_6_ | 459.7842 | 459.7801 |
| 7 | (*N*ae*N*phe*N*phe)_4_ | C_88_H_107_N_17_O_12_ | 797.9221 | 797.9189 |
| 8 | (*N*ae*N*phe*N*phe)_3_ | C_66_H_81_N_13_O_9_ | 600.8218 | 600.8185 |
| 9 | (*N*ae*N*phe*N*phe)_2_ | C_44_H_55_N_9_O_6_ | 806.4354 | 806.437 |
| 10 | (*N*ae*N*spe*N*spe)_4_ | C_96_H_123_N_17_O_12_ | 853.9847 | 853.9841 |
| 11 | (*N*ae*N*spe*N*spe)_3_ | C_72_H_93_N_13_O_9_ | 642.8688 | 642.8675 |
| 12 | (*N*ae*N*spe*N*spe)_2_ | C_48_H_63_N_9_O_6_ | 862.4980 | 862.4994 |

*Table S2. Accurate mass spectrometry data and data from analytical RP-HPLC for representative compounds of this peptoid library.* *Accurate mass data calculated for the [M+2H]^2+^ ion.*

## Representative Analytical RP-HPLC

Analytical HPLC traces for representative peptoids in the library are shown below.

|  | Sequence | RP-HPLC Retention  *(min)* | RP-HPLC Approx. Purity  (%) |
| --- | --- | --- | --- |
| 7 | (*N*ae*N*phe*N*phe)_4_ | 15.2 | >95 |
| 4 | (*N*Lys*N*spe*N*spe)_4_ | 16.2 | >95 |
| 5 | (*N*Lys*N*spe*N*spe)_3_ | 15.3 | >95 |
| 6 | (*N*Lys*N*spe*N*spe)_2_ | 14.4 | >95 |
| 10 | (*N*ae*N*spe*N*spe)_4_ | 16.0 | >95 |

*Table S3. Data from analytical RP-HPLC for representative compounds of this peptoid library.* *Analytical HPLC gradient: 0 – 100% solvent B over 30 min at 220 nm (where solvent A = 95% H_2_O, 5% MeCN, 0.05 % TFA; solvent B = 95% MeCN, 5% H_2_O, 0.03% TFA).*

**Analytical HPLC trace peptoid 7 (*N*ae*N*phe*N*phe)_4_**

**Analytical HPLC trace peptoid 4 (*N*Lys*N*spe*N*spe)_4_**

**Analytical HPLC trace peptoid 5 (*N*Lys*N*spe*N*spe)_3_**

**Analytical HPLC trace peptoid 6 (*N*Lys*N*spe*N*spe)_2_**

**Analytical HPLC trace peptoid 10 (*N*ae*N*spe*N*spe)_4_**

## Chemical and Physical Data for Peptoid Library

LC-MS data, RP-HPLC retention and the approximate yield after purification are tabulated for the peptoid library in *Table S4.* LC-MS spectra are shown below the table; for each peptoid the mass spectrum (top) and UV chromatogram (bottom) are shown.

|  | Sequence | Mass Calculated  *[M+H]+* | Mass Observed  *[M+H]^+^ or *[M+2H]^2+^* | RP-HPLC Retention  *(% MeCN)* |
| --- | --- | --- | --- | --- |
| 1 | (*N*Lys*N*phe*N*phe)_4_ | 1707.2 | 1707.5 | 37 |
| 2 | (*N*Lys*N*phe*N*phe)_3_ | 1284.6 | 1284.7 | 34 |
| 3 | (*N*Lys*N*phe*N*phe)_2_ | 862.1 | 862.5 | 31 |
| 4 | (*N*Lys*N*spe*N*spe)_4_ | 1819.4 | 1819.4 | 42 |
| 5 | (*N*Lys*N*spe*N*spe)_3_ | 1368.8 | 1368.8 | 37 |
| 6 | (*N*Lys*N*spe*N*spe)_2_ | 918.2 | 918.6 | 33 |
| 7 | (*N*ae*N*phe*N*phe)_4_ | 1594.9 | 1595.2 | 37 |
| 8 | (*N*ae*N*phe*N*phe)_3_ | 1201.5 | 1201.0 | 34 |
| 9 | (*N*ae*N*phe*N*phe)_2_ | 806.0 | 806.4 | 30 |
| 10 | (*N*ae*N*spe*N*spe)_4_ | 1708.2 | 1708.0 | 41 |
| 11 | (*N*ae*N*spe*N*spe)_3_ | 1284.6 | 1284.7 | 39 |
| 12 | (*N*ae*N*spe*N*spe)_2_ | 862.1 | 862.5 | 33 |

*Table S4. Calculated and observed masses from LC-MS analysis of the peptoid library tested in this study. All peptoids are amidated at the C terminus. Typical HPLC gradient: 0 – 50% solvent B over 60 min then 50 – 100% solvent B over 25 mins (where solvent A = 95% H_2_O, 5% MeCN, 0.1 % TFA; solvent B = 95% MeCN, 5% H_2_O, 0.1% TFA), % MeCN calculated from middle of elution peak.*

## LC-MS Spectra for Peptoid Library

LC-MS spectra for the peptoid library; for each peptoid the mass spectrum (top) and UV chromatogram at λ = 250 nm (bottom) are shown.

**Peptoid 1 (*N*Lys*N*phe*N*phe)_4_**

**Peptoid 2 (*N*Lys*N*phe*N*phe)_3_**

**Peptoid 3 (*N*Lys*N*phe*N*phe)_2_**

**Peptoid 7 (*N*ae*N*phe*N*phe)_4_**

**Peptoid 8 (*N*ae*N*phe*N*phe)_3_**

**Peptoid 9 (*N*ae*N*phe*N*phe)_2_**

**Peptoid 4 (*N*Lys*N*spe*N*spe)_4_**

**Peptoid 5 (*N*Lys*N*spe*N*spe)_3_**

**Peptoid 6 (*N*Lys*N*spe*N*spe)_2_**

**Peptoid 10 (*N*ae*N*spe*N*spe)_4_**

**Peptoid 11 (*N*ae*N*spe*N*spe)_3_**

**Peptoid 12 (*N*ae*N*spe*N*spe)_2_**
